# Patient-reported outcomes item selection for bladder cancer patients in chemo- or immunotherapy

**DOI:** 10.1186/s41687-019-0141-2

**Published:** 2019-08-22

**Authors:** Gry Assam Taarnhøj, Henriette Lindberg, Christoffer Johansen, Helle Pappot

**Affiliations:** 1 Department of Oncology, University Hospital of Copenhagen, Rigshospitalet, Blegdamsvej 9, 2100 Copenhagen, Denmark; 2 Department of Oncology, University Hospital of Copenhagen, Herlev Hospital, Herlev, Denmark

**Keywords:** Patient-reported outcomes, Item selection, Bladder cancer, Chemotherapy, Immunotherapy

## Abstract

**Background:**

Selection of specific patient-reported outcomes (PROs) for cancer patients requires careful consideration to the purpose and population at aim. Here we report the process of choosing which items to include in a bladder cancer population in chemo- or immunotherapy based on the Patient-Reported Outcomes Version of the Common Terminology Criteria of Adverse Events (PRO-CTCAE).

**Methods:**

Initial PRO-CTCAE symptoms were chosen through 1) medical record audit 2) patient interviews 3) summary of product characteristics from European Medicines Agency and Food and Drug Administration for the applied chemotherapies, and 4) toxicity reporting from Phase 2 and 3 trials for immunotherapies applied in patients with urothelial cancer. The selected questions were applied in a prospective cohort of 78 bladder cancer patients receiving chemo- or immunotherapy at Rigshospitalet and Herlev Hospital, Denmark. Symptoms tested in this population were selected for the final module if they appeared in ≥3 of the following groupings a) the most prevalent PRO-CTCAE symptoms grade ≥ 2 overall during treatment b) the PRO-CTCAE symptoms reported in conjunction with hospital admissions or mentioned in focus group interviews discussing which symptoms were prevalent in this patient group with specialized c) nurses or d) physicians. The authors also included symptoms in the final module if they were present in two of the above groups *and* defined as actionable by clinicians.

**Results:**

From the initial selection of PRO-CTCAE symptoms, a total of 45 PRO-CTCAE symptoms explored by 84 PRO-CTCAE questions were retrieved. Through the second selection process based on the described criteria, the study group agreed on 15 PRO-CTCAE symptoms explored by 30 PRO-CTCAE items to be appropriate and relevant for the bladder population during medical oncological treatment.

**Conclusions:**

The selection of disease specific PROs in a bladder cancer population was feasible. The process revealed several steps of selection needed in order to reach a final module for clinical application.

**Electronic supplementary material:**

The online version of this article (10.1186/s41687-019-0141-2) contains supplementary material, which is available to authorized users.

## Background

For decades toxicity monitoring during treatment for cancer has been a priority in clinical cancer care practice. The Common Terminology Criteria of Adverse Events (CTCAE) has enabled uniform clinician reporting of toxicities across clinical trials and countries [1]. However, an increasing body of literature informs us of the incongruence between patient and clinician reporting. The clinician typically underscores the symptomatic adverse events of the patient thereby underestimating the effect a given symptom has on the patient’s life [2, 3]. To overcome this gap between patient and clinician, the National Cancer Institute (NCI) in the US developed the Patient-Reported Outcomes version of Common Terminology Criteria of Adverse Events (PRO-CTCAE) as a supplement to regular toxicity monitoring [4]. The introduction of patient-reported outcomes (PROs) in cancer care as a proactive symptom management tool has in clinical trials shown to be statistically and clinically advantageous for the patient in terms of better quality of life and even survival [5–9]. Since 2009 the US Food and Drug Administration (FDA) has advocated for the incorporation of PROs in clinical trials [10]. Despite the FDA recommendation, the NCI initiative and the reporting of clinical benefits, the use of the PRO-CTCAE or other PRO tools for symptom monitoring is not a standard along with the CTCAE reporting in daily clinical practice. The reasons for this may be multiple. One of the reasons, as hypothesized by the authors, may be hesitation and awareness of choosing the right questions to ask [11]. A given collection of items may rule out other items that for some patients are central. Important to have in mind is the aim of applying PROs, thus defining a subset of questions crucial for timely capture of progressing symptoms important to the aim. Questions relevant for capturing early relapses in a survivorship setting may be different from the questions relevant for sustaining an acceptable quality of life during treatment. The questions will therefore differ depending on the population at hand hence requiring careful thought and knowledge of both population and aim. The process of defining which questions to ask may therefore delay the implementation of PROs across cancer diseases. For cancer patients with comorbidities and limited treatment options implementation of PROs can be a safe and toxicity-free way of improving survival [5, 9]. One such population is bladder cancer patients known to deal with several comorbidities [12, 13]. Patients with muscle-invasive disease (T2-) or metastases have poor clinical outcomes [14–16] and comorbidities trouble treatment completion thus impairing prognosis. We have in a previously conducted study observed that 31% of bladder cancer patients experience symptomatic toxicities leading to hospital admissions or discontinuation of treatment (24%) [17]. Patient-reported outcomes may increase patient awareness towards symptoms and their management, thereby affecting treatment adherence and in this way be an option for these patients to improve clinical outcomes.

The aim of this study was to define the relevant PRO-CTCAE items for a PRO instrument applicable in a population of bladder cancer patients in medical oncological treatment. The final PRO instrument was planned to be applied in a randomized trial (NCT03584659) with co-primary endpoints of increasing completion of treatment and reducing hospital admissions by intervening with this PRO instrument thus allowing for application of the described methods in other cancer populations.

## Methods

Data was collected in two stages in this mixed-methods study, applying the results of stage 1 in the methods of stage 2 (see below). The methods applied were inspired by Nissen et al. [11] and comprised medical record audit, patient interviews, literature and medical authorities document review, prospective collection of PROs in a clinical setting and analysis hereof and focus group interviews.

**Fig. 1:**
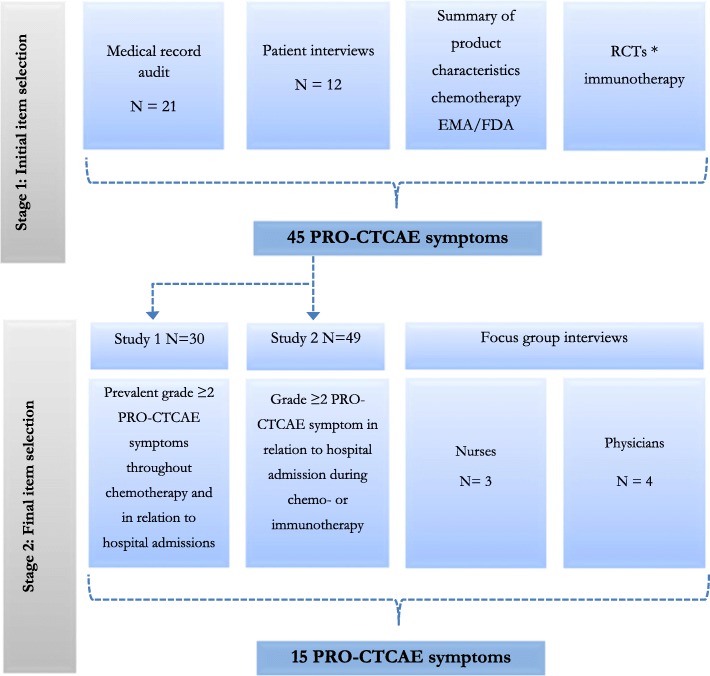
Item Selection Process. legend: *Rosenberg et al., Lancet 2016, Bellmunt et al., NEJM, 2017

The two stages covered the following and are graphically displayed in Fig. 1:

1. Stage:
  - Pilot study:
    i. Medical record audit
    ii. Patient interviews
    iii. Summary of Product Characteristics for the applied chemotherapies (see text below)
    iv. Reported adverse events from Phase 2 and 3 clinical trials of immunotherapy in this population
2. Stage
  - Study 1: Prospective study in the bladder cancer population with collection of PROs and clinical outcomes
  - Study 2: Prospective study in the bladder cancer population with collection of PROs and focus on the feasibility of electronic reporting
  - Study 3: Focus group interviews
    i. Nurses
    ii. Physicians

The *first stage* applied methods introduced by Nissen et al. [11] and included a pilot study for initial item selection:

### Pilot study

Initial selection of PRO-CTCAE items by a process of i) medical record audit was performed until data saturation from 21 consecutive patients receiving chemotherapy for urothelial cancer at Rigshospitalet, Denmark, from January 2017 to April 2017 noting all symptoms documented by physician/nurse during the treatment ii) patient interviews with patients receiving chemotherapy (combination cis- or carboplatin and gemcitabine, or single agent vinflunine) for urothelial cancer until data saturation noting all symptoms mentioned by the patient without regard to severity or frequency. The patients were all asked the same questions according to a predefined questionnaire set and were all informed about the purpose of the interview iii) Summary of Product Characteristics from European Medicines Agency (EMA) and Food and Drug Administration (FDA) for the applied chemotherapies (as mentioned above) noting all symptoms with a frequency of ≥10% of patients iv) toxicity-reporting from Phase 2 and 3 clinical trials of PD1/PDL-1 inhibitors (as this period came prior to marketing of immunotherapies for urothelial cancer, no FDA or EMA Summary of Product Characteristics were available) [18, 19].

All symptoms, regardless of frequency or severity from interviews and medical record audit, from the above process 1a, i-iv, were listed systematically by Medical Dictionary for Regulatory Activities (MedDRA) and aligned with the possible corresponding PRO-CTCAE items thus comprising the initial selection of PRO-CTCAE questionnaire frame (PRO-CTCAE QF1). This initial process is listed in Additional file 1 and graphically displayed in Fig. 1.

For the *second stage*, the results of the pilot study were applied in two prospective clinical studies. The results of these studies were combined with focus group interviews to conclude the final selection:

### Study 1. Patient-reported outcomes and clinical outcomes

Prospective clinical study of 30 patients receiving chemotherapy (combination cis- or carboplatin and gemcitabine, or single agent vinflunine) at Rigshospitalet, Denmark, for urothelial cancer (with locally advanced, recurrent or metastatic disease) reporting quality of life (QoL) and selected patient-reported outcomes. All patients reported the following questionnaires weekly electronically or by paper: EORTC QLQ-C30, EORTC QLQ-BLM30, HADS, PRO-CTCAE QF1 and 3 general health questions, a total of 161 questions weekly by each patient throughout the course of chemotherapy. The study period was August 2017 – August 2018. Reporting ceased when terminating treatment, for whatever reason, or if the patient withdrew consent. Clinical data for all patients were documented: completion of treatment, reason for discontinuation of treatment, hospital admissions and reasons for these. Results from this study have been reported in a separate paper [17]. For the current study all PRO-CTCAE symptoms grade ≥ 2 (regardless of which PRO-CTCAE item (frequency, severity, interference with daily activities) were scored grade ≥ 2, PRO-CTCAE scale is 0–4) throughout the course of chemotherapy and in relation to all hospital admissions were listed.

### Study 2: feasibility of electronic reporting in a co-morbid population

Prospective clinical study of 49 patients with locally advanced, recurrent or metastatic disease receiving chemo- (combination cis- or carboplatin and gemcitabine, or single agent vinflunine) or immunotherapy (pembrolizumab) and reporting symptoms and QoL by the same questionnaire frame as in study 1, weekly throughout the course of treatment. All patients in this study completed weekly reporting electronically and the study was carried out at two university hospitals, Rigshospitalet and Herlev Hospital. The study period was February 2018 – January 2019. The purpose of this study was primarily testing of feasibility of electronic reporting and enrolment at two oncological centres prior to initiation of the planned randomized trial. For the current study, all PRO-CTCAE symptoms grade ≥ 2 data in conjunction with hospital admissions (completed questionnaires within one week of day of admission to hospital as the PRO-CTCAE questionnaire time frame is one week) for patients receiving chemo- or immunotherapy were retrieved.

### Study 3: focus group interviews

Two focus group interviews were conducted, with specialist uro-oncological nurses (*n* = 3) and physicians (*n* = 4), separately. Only eligibility criterium was > 2 years of clinical experience with bladder cancer patients. The focus groups took place in August 2018. Both groups were asked to list symptoms experienced by the group of urothelial cancer patients, regardless of relation to treatment, and both groups were posed the same questions regarding the patient group by author GAT. Both focus group interviews were recorded and followingly transcribed. All symptoms from these interviews were listed, irrespective of frequency or severity. No upper limit in length of the interviews was predefined and the clinicians were encouraged to talk about the subject until group agreement on saturation of data.

Symptoms extracted from stage 2 (studies 1–3) above were listed in four columns, as displayed in Table 1 and Fig. 1. All symptoms mentioned in three or more columns were included in the final questionnaire frame. Symptoms mentioned in two columns were included if the given symptom was preventable/treatable or in some way could be handled by clinical staff, as evaluated by the study group. This final selection process was conducted by the study group in October 2018.

**Table 1:** Patient characteristics from study 1 and 2

Clinical data		Total *n* = 79 (%)	Study 1 *N* = 30 (%)	Study 2 *n* = 49 (%)
Gender	Men	64 (81%)	22 (73%)	42 (86%)
	Women	15 (19%)	8 (27%)	7 (14%)
Median age, yrs. (range)		68 (35–82)	68 (35–82)	68 (48–80)
Stage	Locally advanced	26 (33%)	13 (43%)	13 (27%)
	Metastatic	53 (67%)	17 (57%)	36 (73%)
Treatment^a^	Cisplatin + gemcitabine	46 (59%)	22 (76%)	24 (49%)
	Carboplatin + gemcitabine	9 (11%)	6 (21%)	3 (6%)
	Vinflunine	3 (4%)	1 (3%)	2 (4%)
	Pembrolizumab	20 (26%)	0 (0%)	20 (41%)
Admission to hospital	No	32 (41%)	10 (34%)	22 (45%)
	Yes	46 (59%)	19 (66%)	27 (55%)

^a^One patient in study 1 never started treatment due to cerebral stroke before initiation of treatment

## Results

In the initial pilot study, the four processes, i-iv, revealed a total of 84 symptoms, listed by Medical Dictionary for Regulatory Activities (MedDRA) system organ class. These 84 symptoms corresponded to 45 PRO-CTCAE symptoms explored by 84 PRO-CTCAE items, as many PRO-CTCAE symptoms are explored by single items on frequency, severity and interference with daily activities. The symptoms not corresponding to a PRO-CTCAE symptom were found not to do so primarily because of the symptom not being assessable by the patient her/himself, e.g. hyponatremia. As an example, hyponatremia is easily documented in the summary of product characteristics of a given drug, but more difficultly defined by symptomology as e.g. confusion, weakness, shivers, nausea or deliria could be present all at once or as single symptoms and thus detectable by the patient and reportable by other more comprehensive PRO-CTCAE symptoms/items. The results of the pilot study are listed in Additional file 1. The 84 PRO-CTCAE items were defined to be included in study 1 and study 2.

For study 1, 31 patients were approached and asked to participate. Only one patient declined participation due to lack of energy. For study 2, 58 patients were approached and 49 accepted enrolment. The nine patients declining participation were either not comfortable with the IT solution (*n* = 2) or did not want to spend time replying to questionnaires (*n* = 7). The demographics for patients participating in study 1 and 2 aligns well with previous literature, see Table 1 [16]. The clinical course of these patients and reporting of quality of life have been reported in a separate paper [17]. The overall questionnaire completion rate was high (Study 1: 71%, Study 2: 75%) with a declining tendency over time.

The extraction of all reported PRO-CTCAE symptoms grade ≥ 2 from study 1 resulted in a list of 34 PRO-CTCAE symptoms, listed in falling frequency in Table 2. Likewise, by extraction of the grade ≥ 2 PRO-CTCAE symptoms in relation to hospital admissions in study 2 resulted in 21 PRO-CTCAE symptoms, aligned in Table 2 with the results from study 1.

**Table 2:** Final PRO-CTCAE Item Selection Process

Focus group interviews		Overall PRO-CTCAE symptoms grade 2-4 for 29 patients* (listed by falling frequency, %)	PRO-CTCAE symptoms grade 2-4 in relation to hospital admission (patients receiving n=13 chemo- / n=7 immunotherapy)**	Final included PRO-CTCAE symptom
Nurses, n=3	Doctors, n=4			
Urinary symptoms	Urinary symptoms/catheter problems/infections	Frequent urination (48)	Frequent urination (2)	Frequent urination
Fatigue	Fatigue	Fatigue (36)	Fatigue (9)	Fatigue
		Urinary urgency (30)		
		Dry mouth (25)	Dry mouth (2)	
Nausea	Nausea	Nausea (22)	Nausea (4)	Nausea
Pain	Pain	General pain (22)	General pain (7)	Pain
Decreased appetite	Decreased appetite	Decreased appetite (21)	Decreased appetite (4)	Decreased appetite
Swelling	Swelling	Swelling (21)	Swelling (5)	Swelling
	Insomnia	Insomnia (20)	Insomnia (5)	Insomnia
	Shortness of breath	Shortness of breath (19)	Shortness of breath (5)	Shortness of breath
	Constipation	Constipation (15)	Constipation (1)	Constipation
	Abdominal pain	Abdominal pain (15)	Abdominal pain (5)	
		Sad (15)		
	Heart palpitations	Heart palpitations (14)	Heart palpitations (1)	Heart palpitations
		Painful urination (13)		
		Muscle pain (12)	Muscle pain (3)	
Taste changes		Taste changes (12)	Taste changes (3)	
		Decreased libido (11)		
		Urinary incontinence (11)		
	Diarrhea	Diarrhea (11)	Diarrhea (4)	Diarrhea
		Chills (11)	Chills (1)	Chills
		Cough (10)		
		Headache (10)		
	Neuropathy	Numbness & tingling (9)		
		Dry skin (9)	Dry skin (1)	
		Discouraged (8)		
		Ringing in ears (8)		
		Itching (7)	Itching (1)	Itching
Anxiety		Anxious (6)		Anxious
		Dizziness (5)		
		Difficulty swallowing (5)		
		Increased sweating (4)		
		Mouth /throat soars (4)		
		Joint pain (4)	Joint pain (1)	
		Heartburn (3)		
		Blurred vision (3)		
		Hair loss (3)		
		Hot flashes/flushes (2)		
	Vomiting	Vomiting (2)		Vomiting
		Memory (1)	Memory (1)	
		Concentration (1)	Concentration (1)	
	Weight loss			
	Stomatitis			

^a^Of the 30 patients enrolled in study 1, only 29 initiated treatment

^b^Of the 27 patients in study 2 experiencing hospital admission, only 20 (13 chemotherapy / 7 immunotherapy) patients had a completed questionnaire in relation to the hospital admission

The focus group interviews in study 3 had a duration of 30–45 min. All mentioned symptoms during the focus groups were included and resulted in eight symptoms included from the focus group interview with specialist nurses and 16 symptoms from the focus group with uro-oncologists. All symptoms yielded from study 1–3 in stage two are listed and aligned in Table 2.

Through the selection process described in the methods section a total of 15 PRO-CTCAE symptoms explored by 30 PRO-CTCAE questions were selected for weekly reporting in the planned randomized trial. Illustrating an example of this process is the PRO-CTCAE item on chills which was only present in two out of four of the defined columns in Table 2. This symptom was however found manageable by clinicians as a possible sign of fever and infection and therefore included in the final set despite only being present in two columns. To restrain the amount of questions two exceptions to the inclusion criteria above were made to this framework; regarding the PRO-CTCAE item on taste changes that was mentioned in three of four columns in Table 2. However, the study group found this item irrelevant for weekly reports and thereby possible weekly interventions in the following randomized trial. Also, the PRO-CTCAE item on abdominal pain was by the study group found to be covered by the PRO-CTCAE item on general pain and was therefore not included in the final set of PRO-CTCAE symptoms. No further adjustments were made to limit the volume of the final questionnaire after going through the processes described above.

The final PRO-CTCAE symptoms included are marked with bold font in Table 2 and consist of frequent urination, fatigue, nausea, decreased appetite, pain, shortness of breath, constipation, swelling, insomnia, heart palpitations, chills, anxiety, itching, vomiting and diarrhea.

No data from non-responders was available since the reasons for non-response typically were deterioration of performance status, hospital admissions or death.

## Discussion

We identified 15 symptoms to be explored weekly by 30 single PRO-CTCAE items among bladder cancer patients in oncological treatment when applying PRO-CTCAE items on frequency, severity and/or interference with daily activities, as validated by the National Cancer Institute. The framework of this selection process is anticipated transferable to other cancer populations and the authors encourage application of these methods in future item selection processes.

A similar systematic approach to the selection process of PRO-CTCAE items has recently been published by Trask et al. [20]. The focus is here the selection of PRO-CTCAE items for industry-sponsored clinical trials thus considering e.g. consensus decisions from industry-based working groups. Trask et al. sketch a case example of item selection for a phase 3 trial in metastatic castrate-resistant prostate cancer, not too unfamiliar to the bladder cancer population [20]. This multi-layered selection process is not unlike the process proposed in the present study, however, due to the aim in industry-sponsored trials little attention is paid to the patients’ and caregivers’ perspectives. This discrepancy in methods between Trask and the present study illustrates clearly the importance in choice of methods relevant to the aim and population as mentioned initially. Especially this is relevant when applying PROs for proactive symptom management with the aim of improving HRQOL (health-related quality of life) or overall survival. Application of a given set of PROs developed for capture of e.g. symptoms related to cessation of treatment or hospital admissions in a population of bladder cancer patients may not have the desired effect in a different cancer population (or even the same population) if the aim is increasing quality of life. Much alike, we would not apply the same oncological treatment to bladder and breast cancer patients and expect the same outcome. The objective of this study was, besides the selection of relevant items for a specific population, to describe the process to enable further implementation in similar studies or even in clinical practice. Thus, involvement of patients and clinicians may secure greater success when looking to daily clinical implementation. This idea is supported by Schmidt et al. who describes a process, similar to the current, of both patient and clinician involvement in six focus groups (*n* = 39 health care providers) and by clinical testing (*n* = 71 patients) with the precise aim of implementation in oncological clinical practice. Schmidt et al. found the involvement of patients and health care providers essential for suitable and appropriate selection of PROs and even discovered heterogeneity across participating centres hence recommending interdisciplinary focus groups in each setting [21].

Strengths of this study include the mixed methods design allowing contributions from all stakeholders, patients or clinicians. Without patient and clinician involvement, the final questionnaire frame may not be comprehensive for daily use and implementation. Also, the duration of this process stretching over 22 months enabled thorough conduction of each step of the process. This gradual process is especially important when introducing new technologies and work flows into everyday busy clinical practice to ensure the endorsement from clinicians [22]. Likewise, the time in which this study was conducted allowed for inclusion of patients undergoing immunotherapy with a PD-L1 inhibitor, pembrolizumab, in a standard care setting. Thus, the current and previously reported study 1 [17] are among the first to report real-world data from urothelial cancer patients undergoing immunotherapy in a standard care setting [23]. An important note on the length of this study is of course that in larger cancer institutions the described item selection process is possible over a shorter period of time allowing for application of this process without granting up to two years for the planning.

Despite the attempt to deliver a thorough mixed methods approach to the PRO-CTCAE item selection process, some limitations need to be addressed. First, collection and use of real-world PROs as one of the methods in this study seems intuitive and in respect with the patients exposed to the final questionnaire product. However, the limited number of patients contributing to study 1 introduce the risk of achieving data with a low degree of reliability. This consideration also has relevance in relation to the focus groups including only a limited number of specialist nurses and physicians. To some extent, this is addressed by the mixed methods design. One may also add, that the application of the Delphi method in the focus groups would, potentially, have resulted in more reliable data [24]. Also, given the limited number of patients in immunotherapy participating in study 2, the level of information from this part of the process may be insufficient. This issue is sought weighed up by the RCT’s and focus groups given information on patients in immunotherapy but should nevertheless not be overlooked. Last, selection bias in the PRO items included arise from the fact, that a number of patients did not participate and non-responders in survey studies are reported to have a poorer prognosis [25–27]. This group of non-participants may have other preferences/problems compared to participants and we have no information on non-participants.

## Conclusions

This study presents a transferable set of PROs to apply in a bladder cancer population undergoing medical oncological treatment. A methodological approach to item selection is vital for an effect on clinical outcomes and the effects of the chosen PROs in this study will be tested in a forthcoming randomized trial. The systematic selection process displayed here may assist the planning of future research or support clinical implementation in not only the bladder cancer population but across all cancer diseases.

## Additional file

Additional file 1: PRO-CTCAE ITEM SELECTION FOR PILOT STUDY. (DOCX 42 kb)

## Data Availability

The data that support the findings of this study are available from Gry Assam Taarnhøj but restrictions apply to the availability of these data, which were used under license for the current study, and so are not publicly available. Data are however available from the authors upon reasonable request and with permission of Gry Assam Taarnhøj.

## Abbreviations

CTCAE: Common Terminology Criteria of Adverse Events
EMA: European Medicines Agency
EORTC: European Organisation for Research and Treatment of Cancer
FDA: Food and Drug Administration
HADS: Hospital Anxiety and Depression Scale
HRQOL: Health-related quality of life
MedDRA: Medical Dictionary for Regulatory Activities
NCI: National Cancer Institute
PD1/PDL-1: Programmed cell death protein 1/Programmed death-ligand 1
PRO: Patient-reported outcomes
PRO-CTCAE: Patient-Reported Outcomes version of the Common Terminology Criteria for Adverse Events
QLQ-BLM30: EORTC module for muscle-invasive bladder cancer
QLQ-C30: EORTC general core module
QoL: Quality of life
