# Supplementary material for: Patient-reported outcomes item selection for bladder cancer patients in chemo- or immunotherapy
Source: J Patient Rep Outcomes. 2019 Aug 22;3:56. doi: 10.1186/s41687-019-0141-2 (PMC6706489; doi:10.1186/s41687-019-0141-2)
Supplement: Supplementary file 1 — PRO-CTCAE ITEM SELECTION FOR PILOT STUDY. (DOCX 42 kb) [file 41687_2019_141_MOESM1_ESM.docx]

Additional file 1. PRO-CTCAE Item selection for pilot study.

| **MedDRA system organ class** |  | **Summary of product characteristics** | | | | | | | | **Patient interviews N=12** | **Journal audit N=21** | **RCT Immuno-therapy*** | **PRO-CTCAE symptom** | **Final included PRO-CTCAE symptom** |
| --- | --- | --- | --- | --- | --- | --- | --- | --- | --- | --- | --- | --- | --- | --- |
|  | **Symptom** | **Cisplatin** | | **Gemcitabin** | | **Vinflunin** | | **Carboplatin** | |  | **2 pt immuno, 19 pt. chemo** |  |  |  |
|  |  | EMA | FDA | EMA | FDA | EMA | FDA | EMA | FDA |  |  |  |  |  |
| **Metabolism and nutrition disorders** | Appetite loss | X |  | X |  | X | N/A |  |  | X | X | X | **Decreased appetite** | **X** |
|  | Hyperurikemia | X | X |  |  |  |  | X |  |  |  |  |  |  |
|  | Hyponatremia | X | X |  |  | X |  | X | X | X |  |  |  |  |
|  | Hypokalemia |  | X |  |  |  |  | X | X |  | X |  |  |  |
|  | Hyperkalemia |  |  |  |  |  |  |  |  | X | X |  |  |  |
|  | Hypomagnesemia |  | X |  |  |  |  | X | X |  | X |  |  |  |
|  | Hypocalcemia |  | X |  |  |  |  | X | X |  |  |  |  |  |
|  | Hypophosphatemia |  | X |  |  |  |  |  |  |  |  |  |  |  |
|  | Dehydration |  | X |  |  | X |  |  |  | X | X |  |  |  |
|  | Weight loss |  |  |  |  | X |  |  |  | X |  |  |  |  |
|  | Tumorlysis syndrome |  |  |  |  |  |  | X |  |  |  |  |  |  |
|  | Hyperglycemia |  |  |  |  |  |  |  |  |  |  | X |  |  |
|  | Weight gain |  |  |  |  |  |  |  |  | X | X |  |  |  |
| **Nervous system disorders** | Neuropathy | X | X |  |  |  |  | X | X |  | X |  | **Numbness & tingling** | **X** |
|  | Headache |  |  | X | X | X |  |  |  | X | X |  | **Headache** | **X** |
|  | Trouble Sleeping |  |  | X |  |  |  | X |  | X | X |  | **Insomnia** | **X** |
|  |  |  |  | X(somnolence) | X(PRES) | X(syncope) |  |  |  |  |  |  |  |  |
|  | Eyelid ptosis |  |  |  |  |  |  |  |  |  | X |  |  |  |
| **Eye disorders** | Blurred vision |  | X | X |  | X |  | X |  |  | X |  | **Blurred vision** | **X** |
|  | Opticus neuritis |  | X |  |  |  |  |  |  |  |  |  |  |  |
|  | Blindness |  |  |  | X |  |  |  |  |  |  |  |  |  |
| **Respiratory, thoracic and mediastinal disorders** | Cough |  |  | X |  |  |  |  |  |  | X |  | **Cough** | **X** |
|  | Dyspnoea |  |  | X | X |  |  | X | X | X | X | X | **Shortness of breath** | **X** |
|  | Rhinitis |  |  | X |  |  |  |  |  |  |  |  |  |  |
| **Cardiac disorders** | Rhythmic disorders | X | X |  |  | X(prolonged QT+tachycardia+chest pain) |  | X(cardiovascular disease) | X |  |  |  |  |  |
|  | Palpitations |  |  |  |  |  |  |  |  |  | X |  | **Heart palpitations** | **X** |
|  | Acute myocardial infarction |  |  |  |  |  |  |  |  |  |  |  |  |  |
| **Gastrointestinal disorders** | Vomiting | X | X | X |  | X |  | X | X | X | X | X | **Vomiting** | **X** |
|  | Nausea | X | X | X |  | X |  | X | X | X | X | X | **Nausea** | **X** |
|  | Diarrhea | X | X | X |  | X |  | X | X | X | X | X | **Diarrhea** | **X** |
|  | Gastrointestinal (perforation/  haemorrhage/  other) |  |  |  |  |  |  |  |  |  |  |  |  |  |
|  | Dyspepsia |  |  |  |  | X |  |  |  | X | X |  | **Heartburn** | **X** |
|  | Abdominal pain |  |  |  |  | X |  | X | X | X | X |  | **Abdominal pain** | **X** |
|  | Mouth lesions/mucositis/altered taste |  |  | X(mucosal ulceration of mouth) |  |  |  |  | X(stomatitis) | X | X |  | **Dry Mouth** | **X** |
|  |  |  |  | X(stomatitis) |  | X(stomatitis) |  | X(stomatitis) |  |  |  |  | **Mouth/throat sores** | **X** |
|  |  |  |  |  |  |  |  |  |  |  |  |  |  |  |
|  | Taste changes |  |  |  |  |  |  |  |  | X | X |  | **Taste Changes** | **X** |
|  | Dysphagia |  |  |  |  | X |  |  |  |  |  |  | **Difficulty swallowing** | **X** |
|  | Constipation |  |  | X |  | X |  | X | X | X | X |  | **Constipation** | **X** |
| **Hepatobiliary disorders** | Hepatic involvement | X | X | X | X | X |  | X | X |  | X | X |  |  |
| **Skin and subcutaneous tissue disorders** | Skin lesions/rash |  | X | X |  | X |  | X |  | X | X | X | **Rash** | **X** |
|  |  |  |  |  |  |  |  |  |  |  |  |  | Skin dryness |  |
|  |  |  |  | X |  | X |  | X |  | X |  |  | **Itching** | **X** |
|  |  |  |  |  |  | X |  | X |  |  |  |  | **Hives** | **X** |
|  |  |  |  |  |  |  |  |  |  |  |  |  | Hand-foot syndrome |  |
|  | Alopecia |  | X | X |  | X |  | X | X | X | X |  | **Hair loss** | **X** |
|  | Increased sweating |  |  | X |  |  |  |  |  |  |  |  | **Increased sweating** | **X** |
| **Musculoskeletal and connective tissue disordes** | Artralgia |  |  |  |  | X |  | X |  |  |  | X | **Joint pain** | **X** |
|  | Myalgia |  |  |  |  | X |  | X |  | X |  |  | **Muscle pain** | **X** |
|  | Skeletal pain |  |  |  |  | X |  |  |  | X | X |  |  |  |
| **Genereal disorders and administration site conditions** | Fatigue |  | X |  |  | X |  | X | X | X | X | X | **Fatigue** | **X** |
|  | Lesions at i.v. injection | X |  |  |  | X |  | X |  | X | X |  | **Pain and swelling at injection site** | **X** |
|  | Fever and chills | X |  |  |  | X |  |  |  |  |  | X | **Chills** | **X** |
|  | Hot flashes |  |  | X |  |  |  |  |  |  | X |  | **Hot flashes** | **X** |
|  | Influenza-like symptoms |  |  | X |  |  |  | X |  | X |  |  |  |  |
|  | Pain |  |  |  |  |  |  |  | X | X | X |  | **General pain** | **X** |
|  | Capillary-leakage syndrom(universel) |  |  | X | X |  |  |  |  |  |  |  |  |  |
|  | Edemas |  |  |  |  |  |  |  |  | X | X |  | **Swelling** | **X** |
|  |  |  |  |  |  |  |  |  |  |  |  |  |  |  |
| **Ear and labyrinth disorders** | Ototoxicity | X(vestibular toxicity) |  |  |  | X |  | X(impaired hearing) | X |  | X |  | **Dizziness** | **X** |
|  |  | X(impaired hearing) | X |  |  | X(ear pain) |  |  |  |  |  |  |  |  |
|  |  | X(tinnitus) |  |  |  | X |  | X |  | X | X |  | **Ringing in ears** | **X** |
| **Blood and lymphatic system disorders** | Hematological toxicity | X | X | X | X | X |  | X | X | X | X | X |  |  |
| **Renal and urinary disorders** | Dysuria |  |  | X |  |  |  |  | X | X | X |  | **Painful urination** | **X** |
|  |  |  |  |  |  |  |  |  |  | X |  |  | **Urinary urgency** | **X** |
|  |  |  |  |  |  |  |  |  |  | X | X |  | **Urinary frequency** | **X** |
|  |  |  |  | X(hematuria) |  |  |  |  |  | X |  |  | **Change in usual urine colour** | **X** |
|  |  |  |  |  |  |  |  |  |  | X |  |  | **Urinary incontinence** | **X** |
|  | Nephrotoxicity | X | X | X(+HUS) | X |  |  | (X)(+HUS) | X |  | X |  |  |  |
| **Psychiatric disorders** | Trouble thinking or concentrating |  |  |  |  |  |  |  |  | X | X |  | **Concentration** | **X** |
|  |  |  |  |  |  |  |  |  |  | X | X |  | **Memory** | **X** |
|  | Feeling blue and down |  |  |  |  |  |  |  |  | X |  |  | **Discouraged** | **X** |
|  |  |  |  |  |  |  |  |  |  |  | X |  | **Sad** | **X** |
|  | Feeling nervous or anxious |  |  |  |  |  |  |  |  | X | X |  | **Anxious** | **X** |
|  | Appearance |  |  |  |  |  |  |  |  |  |  |  |  |  |
| **Reproductive system and breast disorders** | Impact on sexuality |  |  |  |  |  |  |  |  |  |  |  | Achieve and maintain erection |  |
|  |  |  |  |  |  |  |  |  |  |  |  |  | Ejaculation |  |
|  |  |  |  |  |  |  |  |  |  | X |  |  | **Decreased libido** | **X** |
|  |  |  |  |  |  |  |  |  |  |  |  |  | Delayed orgasm |  |
|  |  |  |  |  |  |  |  |  |  |  |  |  | Unable to have orgasm |  |
|  |  |  |  |  |  |  |  |  |  |  |  |  | Pain w/sexual intercourse |  |
|  | Fertility impairment | X |  | X | X |  |  |  |  |  |  |  |  |  |
|  | Epididymitis (post op.) |  |  |  |  |  |  |  |  |  | X |  |  |  |
| **Infections and infestations** | Infections |  |  |  |  | X |  | X | X |  | X |  |  |  |
| **Immune system disorders** | Allergic reactions | X | X |  |  | X |  | X | X |  |  |  |  |  |
| **Vascular disorders** | Venous thrombosis |  |  |  |  | X |  |  |  | X |  |  |  |  |
|  | Hypertension |  |  |  |  | X |  |  |  | X | X | X |  |  |
|  | Hypotension |  |  |  |  | X |  |  |  |  | X |  |  |  |
| **Injury poisoning and procedural complications** | Carcinogenic potential | X |  |  |  |  |  |  |  |  |  |  |  |  |
|  | Catheter site reactions |  |  |  |  |  |  |  |  |  | X |  |  |  |
|  | Catheter site discharge |  |  |  |  |  |  |  |  | X | X |  |  |  |

* Rosenberg et al, Lancet 2016, Bellmunt et al., NEJM, 2017.

The highlighted rows exhibit the symptoms from the initial item selection process that align with the PRO-CTCAE symptoms which were then used in study 1 and 2.
